# The Repeatedly Elevated Fatty Liver Index Is Associated With Increased Mortality: A Population-Based Cohort Study

**DOI:** 10.3389/fendo.2021.638615

**Published:** 2021-03-12

**Authors:** Chang-Hoon Lee, Kyung-Do Han, Da Hye Kim, Min-Sun Kwak

**Affiliations:** ^1^Department of Internal Medicine, Seoul National University Hospital, Seoul, South Korea; ^2^Department of Statistics and Actuarial Science, Soongsil University, Seoul, South Korea; ^3^Department of Biostatistics, Catholic University of Korea, Seoul, South Korea; ^4^Department of Internal Medicine, Healthcare Research Institute, Healthcare System Gangnam Center, Seoul National University Hospital, Seoul, South Korea

**Keywords:** hepatic steatosis, non-invasive test, mortality, cardiovascular disease, fatty liver

## Abstract

**Aims:**

Non-alcoholic fatty liver disease (NAFLD) has a dynamic disease course, therefore repeated measurements of NAFLD status could have benefits rather than single one. The aim of this study was to investigate the effects of persistent NAFLD on the incidence of myocardial infarction (MI) and stroke and all-cause mortality by using repeated measurement of fatty liver index (FLI).

**Methods:**

About 3 million subjects who had undergone the health screening four times from 2009 until 2013 were included. NAFLD was defined as an FLI ≥60. FLI points were defined as the number of times participants meeting the criteria of NAFLD (0–4). Outcomes included all-cause mortality, MI, and stroke.

**Results:**

The higher the FLI points, the higher the risk of all-cause mortality, MI, and stroke (*P* for trend <0.001, all). Subjects with four FLI points had a higher risk of all-cause mortality (aHR, 1.86; 95% CI, 1.75–1.98; *P* < 0.001), incidence of MI (aHR, 1.3; 95% CI, 1.21–1.40; *P* < 0.001), and stroke (aHR, 1.27; 95% CI, 1.19–1.37; *P* < 0.001) after adjustment for age, sex, smoking, alcohol consumption, income, hypertension, dyslipidemia, diabetes, body mass index, and physical activity. When the 1^st^ and the last FLI were compared, the “incident NAFLD” group had a higher risk for death compared to the “no NAFLD” group (aHR, 1.46; 95% CI, 1.37–1.55), and the “regression of NAFLD” group had a decreased risk for death compared to the “persistent NAFLD” group (aHR, 0.83; 95% CI, 0.77–0.89).

**Conclusion:**

Repeated evaluations of NAFLD status based on FLI measurements could help physicians identify higher-risk groups in terms of mortality, MI, and stroke. The association between FLI worsening or improvement and outcomes also suggests clinical benefits of the prevention and treatment of NAFLD.

## Introduction

Non-alcoholic fatty liver disease (NAFLD), which is the most common chronic liver disease, has a prevalence of 20–30% in the general population, and the prevalence of NAFLD is increasing (1, 2). NAFLD, especially non-alcoholic steatohepatitis, a subtype of NAFLD, or NAFLD with fibrosis, can progress to advanced fibrosis, liver cirrhosis, and hepatocellular carcinoma. In addition, it has been shown that NAFLD is associated with increased risks of diabetes mellitus (DM) (3) and cardiovascular diseases, including myocardial infarction (MI) (4) and stroke (5). Therefore, NAFLD can lead to increased all-cause mortality. However, NAFLD does not always have a progressive disease course. Some ultrasonography (US)-diagnosed NAFLD patients showed regression or remission of fatty liver (6–8). Several studies evaluating the histologic course of NAFLD also showed regression or improvement of NAFLD in some of the sample (9). NAFLD can be considered a dynamic disease; however, there have been no studies showing long-term outcomes of NAFLD with serial evaluations reflecting changes in NAFLD status.

The fatty liver index (FLI) is a well-known surrogate index of fatty liver based on body mass index (BMI), waist circumference (WC), triglycerides (TGs), and γ-glutamyltransferase (GGT) (10). Previous studies have validated its usefulness in predicting US-diagnosed fatty liver (11–13). FLI is widely used in studies (14, 15) and can be measured repeatedly because of its strengths, including its non-invasiveness and simplicity. Therefore, serial measurement of FLI can be an adequate method to reflect dynamic changes in NAFLD. In fact, there are several studies showing the association between a high value for each component of FLI and cardiovascular diseases (16–19). Our hypothesis is that a sustained high FLI, which means a high FLI measured repeatedly, is associated with an increased risk of cardiovascular diseases and mortality.

In this study, we investigated the effects of serial measurements of NAFLD evaluated by FLI on the incidence of cardiovascular diseases, including MI and stroke, and on all-cause mortality based on long-term population data using Korean National Health Insurance Service (NHIS) cohort including more than 3 million people with annual or biennial health checkups. In addition, we also elucidated whether the incidence or regression of NAFLD has any impact on clinical outcomes.

## Methods

### Data Source of National Health Insurance Service

We used the database of the National Health Insurance Service (NHIS), which is managed by the Korean government. Nearly all (97.2% of the Korean population) Koreans are covered by this system (20). The NHIS supports annual or biennial health checkups for all insured Koreans older than 40 years and employees older than 20 years. The NHIS maintains patients’ demographic information, examination data, claims for disease diagnosis codes of the International Classification of Diseases (ICD-10), and treatment information (21).

This study protocol was exempted from review by the Seoul National University Hospital Institutional Review Board because of the retrospective design of the study, and the researchers accessed only de-identified open clinical data for analytical purposes (H-1903-120-1019). The requirement for informed consent from participants was waived because the researchers accessed only de-identified database entries for analytical purposes.

### Study Population

Participants older than 20 years who had undergone the Korean Health Screening in 2012 or 2013 were initially included. Among them, we selected participants who had undergone four health screening examinations from 2009 until 2013, including the last examination in 2012 or 2013. Then, participants with missing data were excluded. Individuals with heavy alcoholism defined as 30 grams/day based on the self-administered questionnaire were also excluded. Participants with chronic liver disease or liver cirrhosis (B15-B19, K70.3, K74.6) at baseline were excluded. Participants who were diagnosed with myocardial infarction (I21, I22) or stroke (I63, I64) or who had a history of heart disease or stroke based on a self-administered questionnaire were additionally excluded. Then, these participants were followed up until December 2017.

### Measurement of Clinical Parameters and Biochemical Analysis

Standardized self-administered questionnaires were collected. The questionnaires included age (years), sex, smoking (never, former, and current), alcohol consumption (frequency and amount), yearly income, regular physical activity, and underlying diseases.

Height (m) and body weight (kg) were measured using an electronic scale, and BMI was calculated as follows: BMI = Body weight (kg)/height^2^ (m^2^). WC was measured at the midpoint between the lower costal margin and the iliac crest by a trained examiner. The systolic blood pressure (SBP) and diastolic blood pressure (DBP) were measured after 5 min of rest.

After overnight fasting, blood samples were collected from each participant and analyzed using a standardized laboratory method. The baseline laboratory examinations included fasting glucose, total cholesterol, low-density lipoprotein cholesterol, high-density lipoprotein cholesterol, triglycerides, aspartate aminotransferase (AST), alanine aminotransferase (ALT), and GGT.

The diagnoses of hypertension, DM, and dyslipidemia were defined using laboratory and anthropometric measurement data (systolic blood pressure 140 mmHg or diastolic blood pressure 90 mmHg; fasting glucose level ≥126 mg/dl; total cholesterol levels ≥240 mg/dl) or ICD codes (ICD I10 to I13 or I15; E11 to E14; E78) and medication use, including antihypertensive medication, insulin or oral hypoglycemic agents, or dyslipidemia medication.

### Non-Invasive Index of Non-Alcoholic Fatty Liver Disease and Definition of FLI Points

NAFLD was defined according to the well-validated non-invasive FLI in patients without other chronic liver diseases (10, 22). The FLI was calculated as follows:

$$\begin{array}{l} {\text{FLI}}^{10}=(e^{0.953\times \text{Ln}\;(\text{triglycerides})+0.139*\text{BMI}+0.178*\text{Ln}\;(\text{GGT})+0.053\times \text{waist}\;\text{circumference}\;-\;15.745)}\\ /(1+e^{0.953\times \text{Ln}\;(\text{triglycerides})+0.139\times \text{BMI}+0.718\times \text{Ln}\;(\text{GGT})+0.053\times \;\text{waist}\;\text{circumference}\;-\;15.745})\times 100 \end{array}$$

**Figure 1:**
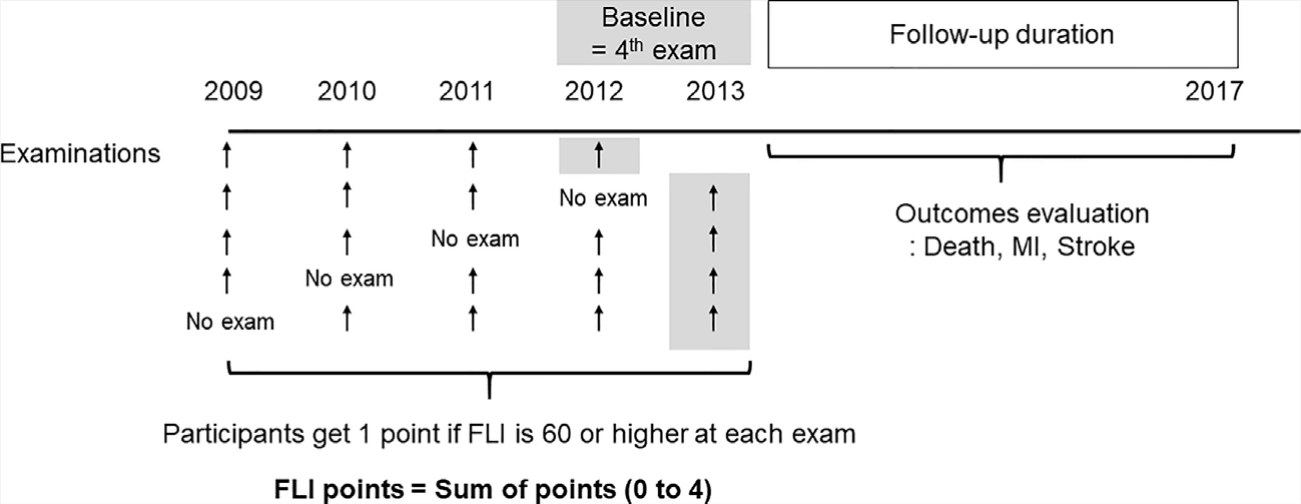
Definition of the fatty liver index (FLI) points and study design. The participants get one point if FLI is 60 or higher at each exam. The FLI points were defined as sum of points for four examinations.

Based on previous studies, participants with FLI < 60 were categorized as having a low likelihood of NAFLD, and those with FLI ≥ 60 were categorized as having a high likelihood of NAFLD (10, 23). To show easily the changing status of NAFLD on serial measurements, we defined “FLI points” as the number of meeting the criterion of NAFLD in 4 serial exams (0–4). Participants received 1 point at each measurement if the FLI was 60 or more. Participants who had FLI ≥ 60 in all four exams (at 2009, 2010, 2011, 2012, or 2013) received 4 FLI points and participants who had FLI < 60 at all four exams received 0 FLI points (**Figure 1**).

In addition, according to the 1^st^ and the last measurements of FLI, we also divided participants into four groups: the “no NAFLD” group (FLI < 60 at the 1^st^ and last exams), “persistent NAFLD” group (FLI ≥ 60 at 1^st^ and last exams), “incident NAFLD” group (FLI < 60 at 1^st^ and FLI ≥ 60 at last), and “regression of NAFLD” group (FLI ≥ 60 at 1^st^ and FLI < 60 at last).

### Outcomes

We evaluated the incidence of MI and stroke using the claim records of NHIS during the follow-up period. MI was defined when ICD code I21 or I22 was recorded with hospitalization. Stroke was diagnosed by the ICD code I63 or I64 with hospitalization and when a claim for imaging study including computed tomography or magnetic resonance was made (24). The NHIS database also provides the date of death if the participants died.

### Statistical Analysis

Continuous variables are expressed as the means ± standard deviations, and categorical variables are expressed as numbers and percentages. Group comparisons were performed using Student’s t-test or one-way analysis of variance for continuous variables and Chi-square tests for categorical variables. For non-normally distributed variables, log transformation was performed.

The incidence rates of MI, stroke, and all-cause mortality were calculated as the number of events divided by total person-years (per 1,000). To adjust for covariates, multivariable Cox proportional hazards regression models were used. We also performed subgroup analysis to evaluate the impact of FLI on mortality according to the subgroups.

Statistical analyses were performed using SAS version 9.3 (SAS Institute Inc., Cary, NC, USA) and R version 3.2.3 (The R Foundation for Statistical Computing, Vienna, Austria). A two-sided *P* value less than 0.05 was considered statistically significant.

## Results

The flowchart of the study is presented in **Supplementary Figure 1**. Of 3,003,068 participants, 2,380,050 participants (79.3%) never had an FLI ≥ 60 in the four consecutive tests (FLI points = 0, no NAFLD), 210,204 (7.0%) had 1 FLI point, 131,610 (4.4%) had 2 FLI points, 116,118 (3.9%) had 3 FLI points, and 165,086 (5.5%) had 4 FLI points. The baseline characteristics of participants according to FLI points are presented in **Table 1**, which shows that the higher the FLI points, the higher the proportion of males, current smokers, and alcohol consumers. Those with higher FLI points had worse health indices, including higher BMI, WC, SBP, DBP, fasting glucose, total cholesterol, and TGs; lower HDL cholesterol; and higher likelihood of hypertension, dyslipidemia, and DM, than those with lower FLI points. Those with higher FLI points had higher levels of AST, ALT, GGT, and FLI than those with lower FLI points at baseline.

**Table 1:** Baseline characteristics according to FLI points.

	Total	FLI point(s)					P- value
		0	1	2	3	4	
Number	3,003,068	2,380,050	210,204	131,610	116,118	165,086	
Age, years	44.3 ± 11.1	44.1 ± 11.3	45.5 ± 10.9	45.2 ± 10.6	45.0 ± 10.3	44.4 ± 9.6	<0.001
Age ≥65 years	136843 (4.6)	110082 (4.6)	10909 (5.2)	6097 (4.6)	4715 (4.1)	5040 (3.1)	<0.001
Men	2012289 (67.0)	1441261 (60.6)	184798 (87.9)	120362 (91.5)	108449 (93.4)	157419 (95.4)	<0.001
Smoking							<0.001
Non-smoker	1552000 (51.7)	1369330 (57.5)	71432 (34.0)	39448 (30.0)	31944 (27.5)	39846 (24.1)	
Ex-smoker	569124 (19.0)	408345 (17.2)	54872 (26.1)	35063 (26.6)	30784 (26.5)	40060 (24.3)	
Current	881944 (29.4)	602375 (25.3)	83900 (39.9)	57099 (43.4)	53390 (46.0)	85180 (51.6)	
Mild alcohol drinking	1717013 (57.2)	1270971 (53.4)	142374 (67.7)	92997 (70.7)	84968 (73.2)	125703 (76.1)	<0.001
Lowest quartile of yearly income (Q1)	668628 (22.3)	553909 (23.3)	41730 (19.9)	24485 (18.6)	20878 (18.0)	27626 (16.7)	<0.001
Regular PA	653225 (21.8)	512196 (21.5)	49583 (23.6)	30796 (23.4)	26844 (23.1)	33806 (20.5)	<0.001
BMI (kg/m^2^)	23.7 ± 3.2	22.8 ± 2.6	25.9 ± 2.4	26.6 ± 2.5	27.4 ± 2.6	29.0 ± 3.0	<0.001
WC (cm)	80.2 ± 8.9	77.8 ± 7.6	86.7 ± 5.9	88.5 ± 6.0	90.9 ± 6.2	94.1 ± 7.0	<0.001
SBP (mmHg)	121.1 ± 13.5	119.4 ± 13.2	125.6 ± 12.9	126.7 ± 12.9	127.7 ± 13.0	129.6 ± 13.2	<0.001
DBP (mmHg)	76.1 ± 9.4	75.0 ± 9.2	79.2 ± 9.1	79.9 ± 9.2	80.7 ± 9.3	82.2 ± 9.6	<0.001
Hypertension	576049 (19.2)	361364 (15.2)	60924 (29.0)	42519 (32.3)	41575 (35.8)	69667 (42.2)	<0.001
Dyslipidemia	498694 (16.6)	314251 (13.2)	52735 (25.1)	36741 (27.9)	35986 (31.0)	58981 (35.7)	<0.001
Diabetes							<0.001
No	2135786 (71.1)	1798185 (75.6)	125383 (59.7)	73674 (56.0)	61100 (52.6)	77444 (46.9)	
IFG	675390 (22.5)	476939 (20.0)	62408 (29.7)	41254 (31.4)	37840 (32.6)	56949 (34.5)	
DM	191892 (6.4)	104926 (4.4)	22413 (10.7)	16682 (12.7)	17178 (14.8)	30693 (18.6)	
Fasting glucose (mg/dl)	96.1 ± 19.9	94.0 ± 16.9	100.7 ± 23.5	102.6 ± 25.4	104.5 ± 27.4	108.6 ± 31.8	<0.001
Total cholesterol (mg/dl)	195.1 ± 34.9	192.0 ± 33.7	203.5 ± 35.9	205.4 ± 36.6	207.2 ± 37.3	211.5 ± 38.2	<0.001
LDL (mg/dl)	114.5 ± 31.9	113.4 ± 30.9	118.7 ± 34.1	118.6 ± 34.8	118.2 ± 35.6	117.7 ± 36.8	<0.001
HDL (mg/dl)	55.0 ± 14.8	56.7 ± 14.6	49.7 ± 13.6	48.6 ± 13.2	47.8 ± 12.9	46.6 ± 13.0	<0.001
Triglyceride (mg/dl)	132.7 ± 88.9	111.1 ± 61.8	185.5 ± 106.2	203.1 ± 111.6	222.3 ± 119.0	259.9 ± 136.9	<0.001
AST (IU/L)	25.4 ± 11.1	23.9 ± 9.2	28.6 ± 13.2	30.1 ± 14.5	31.5 ± 15.5	34.6 ± 17.4	<0.001
ALT (IU/L)	25.9 ± 18.1	22.2 ± 13.1	34.1 ± 21.9	37.7 ± 24.2	41.0 ± 26.3	47.7 ± 29.6	<0.001
Gammaglutamyl transpeptidase (IU/L)	35.9 ± 39.0	26.9 ± 20.8	53.6 ± 47.4	63.2 ± 55.9	73.2 ± 64.7	94.1 ± 81.3	<0.001
Fatty liver index	27.5 ± 23.9	18.2 ± 14.6	50.0 ± 16.4	59.0 ± 15.5	66.8 ± 14.3	80.0 ± 9.8	<0.001

Categorical variables are expressed as number (%); continuous variables are expressed mean ± standard deviation.

FLI, fatty liver index; PA, physical activity; BMI, body mass index; WC, waist circumference; SBP, systolic blood pressure; DBP, diastolic blood pressure; IFG, impaired fasting glucose; DM, diabetes mellitus; LDL, low-density lipoprotein; HDL, high-density lipoprotein; AST, aspartate aminotransferase; ALT, alanine aminotransferase.

**Figure 2:**
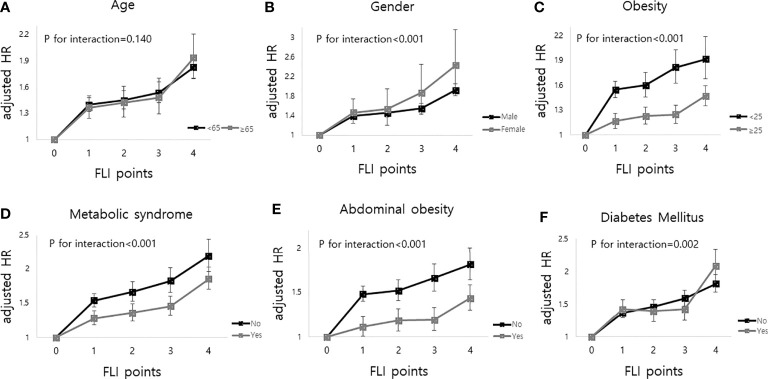
Impact of FLI points on all-cause mortality according to the subgroup analysis as follows; **(A)** age, **(B)** sex, **(C)** obesity (body mass index), **(D)** metabolic syndrome, **(E)** abdominal obesity and **(F)** diabetes mellitus. FLI points had more impacts on all-cause mortality in women, body mass index less than 25, those without metabolic syndrome, those without abdominal obesity, and those with diabetes mellitus.

The median follow-up duration of this cohort was 5.1 years (interquartile range, 4.6–5.5). During follow-up, a total of 20,904 (0.70%, 1.37 incidence rate per 1,000 person years) deaths occurred. MI developed in 13,703 participants (0.46%, 0.90 incidence rate per 1,000 person years), and stroke developed in 14,629 participants (0.49%, 0.96 incidence rate per 1,000 person years). **Table 2** shows that the higher the FLI points, the higher the risk of all-cause mortality, MI, and stroke. Compared to those with 0 FLI points (no NAFLD), the risk of all-cause mortality increased in a dose-dependent manner [adjusted HR (aHR), 1.38; 95% confidence interval (CI), 1.31–1.45 in those with 1 FLI point, aHR 1.44; 95% CI, 1.35–1.53 in those with 2 FLI points, aHR 1.52; 95% CI, 1.42–1.63 in those with 3 FLI points, and aHR 1.86; 95% CI, 1.75–1.98 in those with 4 FLI points, *P* for trend < 0.001] even after adjustment of covariates including age, sex, smoking, alcohol consumption, income, hypertension, dyslipidemia, DM, BMI, and regular physical activity. The risk of MI also increased as the FLI points increased. Compared to those with no NAFLD, those with 4 FLI points had a 1.3-fold increased risk for MI (aHR, 1.30; 95% CI, 1.21–1.40) and a 1.27-fold increased risk for stroke (aHR, 1.27; 95% CI, 1.19–1.37) in the multivariate models. In subgroup analysis, the impact of FLI points on all-cause mortality was more prominent in women, those without obesity, those without metabolic syndrome, and those without abdominal obesity (all *P* for interaction <0.001). Those with DM and higher FLI points also had a higher risk of all-cause mortality than those without DM (*P* for interaction = 0.002). The effect modifications of the subgroups on the association between FLI points and MI and stroke was also observed (**Figure 2**, **Supplementary Table 1**).

**Table 2:** Outcomes including all-cause mortality, myocardial infraction, and stroke according to the FLI points.

	FLI points	n	Events	person-years	Incidence rate per 1,000	Hazard ratio (95% confidence interval)			
						Univariate model	Multivariate model 1	Multivariate model 2	Multivariate model 3
Death	0	2380050	14991	12090315.43	1.23992	1 (reference)	1 (reference)	1 (reference)	1 (reference)
	1	210204	2001	1063627.88	1.8813	1.52 (1.45,1.59)	1.20 (1.14,1.26)	1.38 (1.32,1.45)	1.38 (1.31,1.45)
	2	131610	1230	665650.44	1.84782	1.49 (1.41,1.58)	1.20 (1.13,1.28)	1.44 (1.35,1.53)	1.44 (1.35,1.53)
	3	116118	1083	587186.65	1.84439	1.49 (1.40,1.59)	1.23 (1.16,1.31)	1.53 (1.43,1.63)	1.52 (1.42,1.63)
	4	165086	1599	836355.88	1.91187	1.54 (1.47,1.63)	1.39 (1.32,1.46)	1.87 (1.76,1.99)	1.86 (1.75,1.98)
P for trend						<0.001	<0.001	<0.001	<0.001
MI	0	2380050	9169	12071853.14	0.75954	1 (reference)	1 (reference)	1 (reference)	1 (reference)
	1	210204	1407	1060729.87	1.32645	1.75 (1.65,1.85)	1.45 (1.37,1.54)	1.21 (1.14,1.29)	1.21 (1.14,1.29)
	2	131610	950	663669.71	1.43143	1.89 (1.77,2.02)	1.59 (1.49,1.70)	1.26 (1.17,1.35)	1.26 (1.17,1.35)
	3	116118	839	585386.27	1.43324	1.89 (1.76,2.03)	1.62 (1.51,1.74)	1.22 (1.13,1.32)	1.22 (1.13,1.32)
	4	165086	1338	833439.1	1.6054	2.12 (2.00,2.24)	1.92 (1.81,2.04)	1.31 (1.22,1.40)	1.30 (1.21,1.40)
P for trend						<0.001	<0.001	<0.001	<0.001
Stroke	0	2380050	10167	12068649.6	0.84243	1 (reference)	1 (reference)	1 (reference)	1 (reference)
	1	210204	1441	1060476.91	1.35882	1.61 (1.53,1.71)	1.37 (1.30,1.45)	1.17 (1.10,1.24)	1.17 (1.10,1.24)
	2	131610	946	663529.35	1.42571	1.69 (1.59,1.81)	1.49 (1.39,1.59)	1.22 (1.13,1.31)	1.21 (1.13,1.30)
	3	116118	827	585427.52	1.41264	1.68 (1.56,1.80)	1.53 (1.42,1.64)	1.19 (1.10,1.29)	1.19 (1.10,1.28)
	4	165086	1248	833585.45	1.49715	1.78 (1.68,1.89)	1.78 (1.67,1.89)	1.28 (1.19,1.37)	1.27 (1.19,1.37)
P for trend						<0.001	<0.001	<0.001	<0.001

FLI, fatty liver index; MI, myocardial infarction.

Multivariate model 1 was adjusted for age and sex.

Multivariate model 2 was adjusted for age, sex, smoking, drinking, income, hypertension, dyslipidemia, diabetes, and body mass index.

Multivariate model 3 was adjusted for age, sex, smoking, drinking, income, hypertension, dyslipidemia, diabetes, body mass index, and regular physical activity.

**Figure 3:**
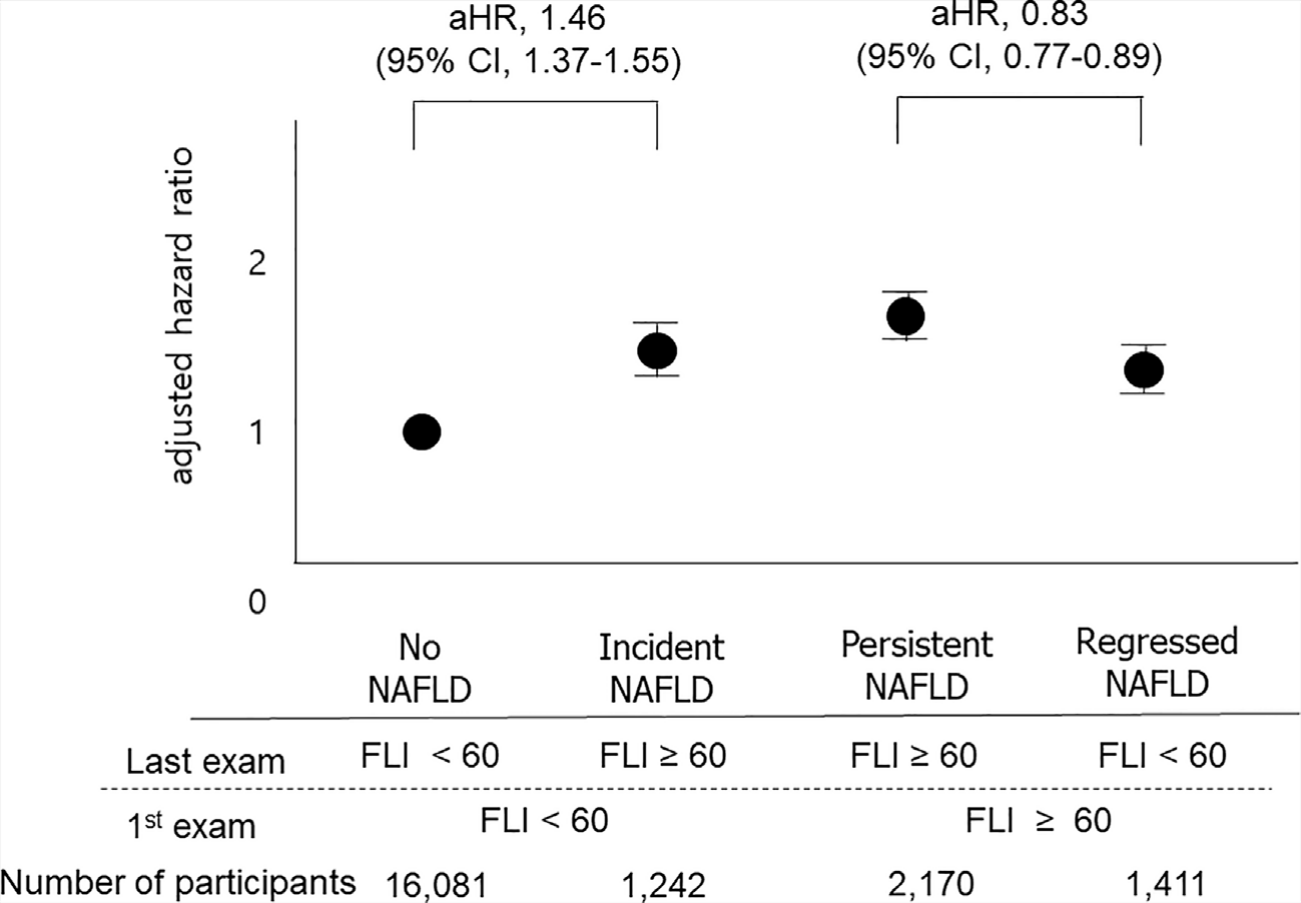
Impacts of changes in FLI between the 1^st^ exam and the last exam on outcomes. The “No NAFLD” group included those with FLI < 60 at both the 1^st^ exam and the last exam, and the “persistent NAFLD” group included those with FLI ≥ 60 at both the 1^st^ exam and the last exam. The “Incident NAFLD” group included those with FLI < 60 at the 1^st^ exam and FLI ≥ 60 at the last exam, and the “Regression of NAFLD” group included those with FLI ≥ 60 at the 1^st^ exam and FLI < 60 at the last exam. Participants with incident NAFLD had a 1.47-fold increased risk for mortality compared to those without NAFLD, and participants with regression of NAFLD had a 0.83-fold decreased risk for mortality compared to those with persistent NAFLD.

We also classified participants into four groups based on the 1^st^ and the last measurements of FLI: the no NAFLD group, the persistent NAFLD group, the incident NAFLD group, and the regression of NAFLD group, as described above. The persistent NAFLD group had the highest risk of all-cause mortality (aHR compared to the no NAFLD group, 1.64; 95% CI, 1.56–1.73), MI (aHR compared to the no NAFLD group, 1.23; 95% CI, 1.16–1.31), and stroke (aHR compared to the no NAFLD group, 1.23; 95% CI, 1.15–1.30) among these four groups. The incident NAFLD group had higher risks of all-cause mortality (aHR, 1.46; 95% CI, 1.37–1.55), MI (aHR, 1.13; 95% CI, 1.05–1.21), and stroke (aHR, 1.17; 95% CI, 1.09–1.26) than the no NAFLD group. The regression of NAFLD group had lower risks of all-cause mortality (aHR, 0.83; 95% CI, 0.77–0.89) than the persistent NAFLD group (**Figure 3**, **Supplementary Table 2**).

## Discussion

This is the first study showing that persistent NAFLD, defined as a repeatedly elevated FLI from four consecutive exams, is associated with a higher risk of all-cause mortality and incidence of MI and stroke than no NAFLD or intermittent NAFLD in a large-scale population-based cohort study. A dose-response relationship was observed between the number of meeting the criterion of NAFLD on four exams and the outcomes (the higher the FLI points, the worse the outcome). The impact of FLI points on all-cause mortality was more prominent in patients with DM, women, those without obesity (BMI <25), those without metabolic syndrome, and those without abdominal obesity. When we compared the FLI between the 1^st^ exam and the last exam, those with incident NAFLD showed higher risks of cardiovascular diseases and all-cause mortality than those without NAFLD. On the other hand, those with improvement of NAFLD had lower risks of all-cause mortality than those with persistent NAFLD.

With increasing obesity and westernized lifestyle, NAFLD is the most prevalent liver disease, with a prevalence of 13–32% (1), and the NAFLD incidence rate estimate for Asians is 52.45 per 1,000 (2). Moreover, the resolution of NAFLD is not an uncommon phenomenon. Several factors, including weight loss or physical activity, can improve NAFLD (25, 26). One study reported that 17.7% of those with NAFLD experienced a resolution of NAFLD over 5 years (27). Another study showed incident NAFLD in 17.1% of patients and resolution of NAFLD in 22.6% of patients at 5 years follow-up (28). Histologic examination of NAFLD also showed dynamic changes with progression or regression along the full spectrum of NAFLD, including even NASH (non-alcoholic steatohepatitis) and NAFLD with fibrosis (9). These results all support that NAFLD is a dynamic disease, which suggests that the evaluation of the long-term outcome of NAFLD based on NAFLD status at only one point has limitations. Previous studies showed that NAFLD is associated with increased mortality (29, 30) and incident cardiovascular disease (5); however, those studies did not reflect the dynamic status of NAFLD. There have been only a few studies evaluating the effect of changing the status of NAFLD on outcomes. A study was conducted to explore the dynamic change in NAFLD. One hospital-based study including approximately 8,000 participants showed that persistence of NAFLD status was a predictive factor of incident type 2 DM compared to never having NAFLD and intermittent NAFLD status from five consecutive exams (31). Increasing liver fat evaluated by CT scans 6 years apart was associated with incident DM and metabolic syndrome (32). There has been no study on the impact of persistency, incidence, or improvement of NAFLD on all-cause mortality and incident cardiovascular diseases. This is the first large-scale population-based study including over 3 million people with four consecutive exams. Four consecutive exams reflect the change in NAFLD status. In our study, if those with NAFLD were defined as those with an FLI ≥ 60 from at least one exam out of four exams, the prevalence would be 20.7%. Among these individuals, approximately one-third (7.0% of total participants) showed FLI ≥ 60 only one time in at least 3 years. Only one-fourth (5.5% of total participants) were classified into the persistent NAFLD group. Those who showed a high FLI only one time had a significantly higher risk of cardiovascular diseases and all-cause mortality than those with no NAFLD. However, those with persistent NAFLD showed the highest risk of cardiovascular diseases and all-cause mortality, and dose-response relationships were also observed in our study. These findings support the concept that NAFLD is a dynamic disease and suggest the clinical benefit of repeated measurements for the assessment of NAFLD status.

The FLI points had a greater impact on all-cause mortality in patients with DM, females, participants without obesity, those without metabolic syndrome, and those without abdominal obesity. The effects of NAFLD and DM on mortality have been suggested in previous studies. A study including (27, 28) NAFLD patients showed a 2.1-fold increased risk of overall mortality in diabetic patients compared to those without DM (33). Another study including approximately 100,000 DM patients showed a 1.6-fold increase in all-cause mortality in subjects with NAFLD compared to those without NAFLD (34). Our results additionally showed the significant multiplicative interactive effect of DM and FLI points on all-cause mortality. Both diseases are associated with insulin resistance, systemic inflammation, and oxidative distress and could contribute to poor long-term outcomes (35). In addition to the presence of DM, sexually dimorphic aspects of the prognosis of NAFLD were noted in this study. The effect of FLI points on mortality was more prominent in the female subgroup than in the male subgroup. One previous hospital-based cohort study showed that NAFLD was independently associated with all-cause death in women but not in men (36). Although the exact mechanisms explaining sexual differences are unknown, changes in the levels of estrogens and faster loss of subcutaneous adipose tissue in aging women compared with men might be related (36–38). The results of this study consistently showed more prominently increased all-cause mortality in females and additionally represented the effect of the persistency of NAFLD, not only the presence of NAFLD, on all-cause mortality according to sex. Intriguingly, NAFLD had more prominent impacts on poor outcomes among so-called metabolically healthy patients. There have been a few studies on whether patients with lean or non-obese NAFLD have worse clinical outcomes, and those small-sized studies (N = 30,740, 46,641, 109,042 respectively) reported different results. Our population-based large-scale study showed that the association between higher FLI points and an increased risk of all-cause mortality was significantly stronger among non-obese participants or those without metabolic syndrome than among those with obesity or metabolic syndrome (39). One cross-sectional study showed that patients with lean NAFLD had more severe histology than overweight patients (40). A longitudinal study showed more development of severe liver disease in lean NAFLD patients than in non-lean NAFLD patients, suggesting increased liver events as a possible explanation for poor outcomes (41). The concept of the “obesity paradox” in chronic liver diseases is also able to explain our results (42). Another interesting finding is that changes in NAFLD status over 3 to 4 years could lead to significantly different prognoses. The “incident NAFLD” group had a higher risk of cardiovascular diseases and all-cause mortality than the “no NAFLD” group, and the “improvement in NAFLD” group had a lower risk of cardiovascular diseases and all-cause mortality than the “persistent NAFLD” group when the 1^st^ and the last exam was compared. These findings suggest the importance of the prevention and treatment of NAFLD. It has been known that NAFLD can be prevented and is treatable. Lifestyle modifications, including weight reduction, dietary changes, and increased exercise, is the main treatment for most patients with NAFLD because there are currently no approved effective pharmacologic agents (43). One study evaluating NAFLD status at two different points showed that resolution of NAFLD status at follow-up improved lipid profiles (27). Our study also showed that NAFLD can be improved (regression of NAFLD), and improvement of NAFLD is associated with better clinical outcomes. Based on the results of studies, healthcare providers could encourage patients with NAFLD to improve their health behavior.

The limitation of the study is that NAFLD was defined by FLI. The gold standard in the evaluation of NAFLD status is liver biopsy, and liver biopsy can also evaluate NASH. However, this method is very invasive and is hardly used to investigate changes. Liver ultrasonography can be a good option, but it requires a manpower than simple serum test, which is a limitation for applying large general populations. The transient elastography (fibroscan) is a simple, useful, non-invasive method to evaluate hepatic steatosis or fibrosis, however, fibroscan is only available at specialized centers owing to its high cost (44). Among several non-invasive markers of NAFLD including FLI, NAFLD liver fat score, hepatic steatosis index, and lipid accumulation product, we used most widely used FLI in this study (45). The FLI is a simple and accurate surrogate marker of hepatic steatosis and has been well validated in many studies (10–14). The FLI is less invasive and more cost effective; it is based on anthropometric measurements of BMI, WC, and serum levels of TGs and GGT (10). A recent study showed that the FLI predicted new cases of NAFLD (46). Therefore, repeated measurement of FLI can be easily and effectively used for the detection of NAFLD in a large general population.

In conclusion, simple repeated evaluation of NAFLD status based on FLI measurements could help physicians identify higher-risk groups in terms of all-cause mortality, MI, and stroke. The association between FLI worsening or improvement and outcomes also suggests clinical benefits of the prevention and treatment of NAFLD.

## Data Availability Statement

The raw data supporting the conclusions of this article will be made available by the authors, without undue reservation.

## Ethics Statement

This study protocol was exempted from review by the Seoul National University Hospital Institutional Review Board because of the retrospective design of the study, and the researchers accessed only de-identified open clinical data for analytical purposes (H-1903-120-1019). The requirement for informed consent from participants was waived because the researchers accessed only deidentified database entries for analytical purposes.

## Author Contributions

M-SK and C-HL contributed to the conception and design of the study, interpretation of data, and drafting of the manuscript. K-DH and DK contributed to the acquisition of data, statistical analysis, table and figure creation, and critical revision of the manuscript for important intellectual content. The corresponding author attests that all listed authors meet the criteria for authorship and that no others meeting the criteria have been omitted. All authors contributed to the article and approved the submitted version. M-SK is the guarantor.

## Conflict of Interest

The authors declare that the research was conducted in the absence of any commercial or financial relationships that could be construed as a potential conflict of interest.

## Abbreviations

GGT, gamma-glutamyltransferase; DM, diabetes mellitus; NHIS, National Health Insurance Service; ICD, International Classification of Diseases; BMI, body mass index; WC, waist circumference; SBP, systolic blood pressure; DBP, diastolic blood pressure; AST, aspartate aminotransferase; ALT, alanine aminotransferase; aHR, adjusted hazard ratio; CI, confidence interval; NASH, non-alcoholic steatohepatitis.
