# Supplementary material for: The Repeatedly Elevated Fatty Liver Index Is Associated With Increased Mortality: A Population-Based Cohort Study
Source: Front Endocrinol (Lausanne). 2021 Mar 12;12:638615. doi: 10.3389/fendo.2021.638615 (PMC7996574; doi:10.3389/fendo.2021.638615)

**Supplementary Tables**

**Supplementary Table 1.** Impact of FLI points on outcome according to the subgroup analysis including age, sex, body mass index, metabolic syndrome, abdominal obesity and diabetes. Presence of abdominal obesity was defined as waist circumference over 90cm in male and 85cm in female.

|  |  |  | Stroke | | | | Myocardial infarction | | | | Death | | | |
| --- | --- | --- | --- | --- | --- | --- | --- | --- | --- | --- | --- | --- | --- | --- |
| Subgroup | FLI points | n | Events | person-years | Incidence rate per 1000 | HR (95% CI) | Events | person-years | Incidence rate per 1000 | HR (95% CI) | Events | person-years | Incidence rate per 1000 | HR (95% CI) |
| Age |  |  |  |  |  |  |  |  |  |  |  |  |  |  |
| <65 | 0 | 2269968 | 7401 | 11536376.12 | 0.64154 | 1 (reference) | 7537 | 11537203.18 | 0.65328 | 1 (reference) | 10350 | 11552539.77 | 0.8959 | 1 (reference) |
|  | 1 | 199295 | 1094 | 1008283.15 | 1.08501 | 1.16 (1.09,1.24) | 1190 | 1008226.09 | 1.18029 | 1.19 (1.12,1.27) | 1458 | 1010683.16 | 1.4426 | 1.40 (1.32,1.48) |
|  | 2 | 125513 | 754 | 634324.27 | 1.18867 | 1.23 (1.13,1.33) | 810 | 634323.96 | 1.27695 | 1.22 (1.13,1.32) | 925 | 636034.35 | 1.4543 | 1.45 (1.35,1.56) |
|  | 3 | 111403 | 667 | 562791.09 | 1.18516 | 1.17 (1.08,1.28) | 735 | 562661.57 | 1.30629 | 1.17 (1.08,1.28) | 843 | 564233.37 | 1.4941 | 1.54 (1.42,1.66) |
|  | 4 | 160046 | 1047 | 809452.58 | 1.29347 | 1.28 (1.12,1.32) | 1232 | 809133.02 | 1.52262 | 1.25 (1.16,1.35) | 1303 | 811816.69 | 1.605 | 1.82 (1.70,1.96) |
|  | P for Trend |  |  |  |  | <0.001 |  |  |  | <0.001 |  |  |  | <0.001 |
| ≥65 | 0 | 110082 | 2766 | 532273.48 | 5.19658 | 1 (reference) | 1632 | 534649.96 | 3.05246 | 1 (reference) | 4641 | 537775.66 | 8.63 | 1 (reference) |
|  | 1 | 10909 | 347 | 52193.76 | 6.6483 | 1.17 (1.04,1.31) | 217 | 52503.78 | 4.13304 | 1.27 (1.10,1.48) | 543 | 52944.72 | 10.256 | 1.37 (1.24,1.50) |
|  | 2 | 6097 | 192 | 29205.08 | 6.5742 | 1.12 (0.96,1.31) | 140 | 29345.75 | 4.77071 | 1.44 (1.20,1.73) | 305 | 29616.09 | 10.2985 | 1.42 (1.26,1.61) |
|  | 3 | 4715 | 160 | 22636.43 | 7.06825 | 1.18 (1.00,1.40) | 104 | 22724.7 | 4.57652 | 1.37 (1.11,1.69) | 240 | 22953.27 | 10.456 | 1.48 (1.29,1.70) |
|  | 4 | 5040 | 201 | 24132.87 | 8.32889 | 1.39 (1.18,1.63) | 106 | 24306.08 | 4.36105 | 1.31 (1.05,1.63) | 296 | 24539.19 | 12.0623 | 1.93 (1.70,2.20) |
|  | P for Trend |  |  |  |  | <0.001 |  |  |  | <0.001 |  |  |  | <0.001 |
| P for interaction |  |  |  |  |  | 0.085 |  |  |  | 0.107 |  |  |  | 0.140 |
| Sex |  |  |  |  |  |  |  |  |  |  |  |  |  |  |
| Male | 0 | 1441261 | 7267 | 7354935.5 | 0.98804 | 1 (reference) | 6779 | 7356638.1 | 0.92148 | 1 (reference) | 12057 | 7370552.97 | 1.63583 | 1 (reference) |
|  | 1 | 184798 | 1248 | 935068.09 | 1.33466 | 1.16 (1.09,1.23) | 1262 | 935165.21 | 1.34949 | 1.22 (1.14,1.30) | 1843 | 937793.89 | 1.96525 | 1.39 (1.32,1.46) |
|  | 2 | 120362 | 859 | 608177.48 | 1.41242 | 1.22 (1.13,1.32) | 884 | 608254.83 | 1.45334 | 1.27 (1.18,1.37) | 1156 | 610104.21 | 1.89476 | 1.46 (1.37,1.56) |
|  | 3 | 108449 | 761 | 547689.36 | 1.38947 | 1.19 (1.09,1.29) | 786 | 547629.81 | 1.43528 | 1.22 (1.12,1.32) | 1024 | 549310.89 | 1.86415 | 1.55 (1.45,1.66) |
|  | 4 | 157419 | 1182 | 795702.34 | 1.48548 | 1.29 (1.19,1.39) | 1287 | 795512.34 | 1.61783 | 1.32 (1.22,1.42) | 1529 | 798331.15 | 1.91525 | 1.92 (1.80,2.05) |
|  | P for Trend |  |  |  |  | <0.001 |  |  |  | <0.001 |  |  |  | <0.001 |
| Female | 0 | 938789 | 2900 | 4713714.1 | 0.61523 | 1 (reference) | 2390 | 4715215.05 | 0.50687 | 1 (reference) | 2934 | 4719762.46 | 0.62164 | 1 (reference) |
|  | 1 | 25406 | 193 | 125408.82 | 1.53897 | 1.27 (1.08,1.48) | 145 | 125564.67 | 1.15478 | 1.20 (1.01,1.44) | 158 | 125833.99 | 1.25562 | 1.47 (1.24,1.74) |
|  | 2 | 11248 | 87 | 55351.88 | 1.57176 | 1.18 (0.94,1.47) | 66 | 55414.88 | 1.19102 | 1.13 (0.87,1.45) | 74 | 55546.24 | 1.33222 | 1.53 (1.20,1.95) |
|  | 3 | 7669 | 66 | 37738.16 | 1.74889 | 1.27 (0.98,1.64) | 53 | 37756.47 | 1.40373 | 1.27 (0.95,1.69) | 59 | 37875.76 | 1.55772 | 1.87 (1.42,2.45) |
|  | 4 | 7667 | 66 | 37883.11 | 1.7422 | 1.24 (0.95,1.62) | 51 | 37926.76 | 1.3447 | 1.14 (0.84,1.54) | 70 | 38024.74 | 1.84091 | 2.43 (1.87,3.16) |
|  | P for Trend |  |  |  |  | 0.008 |  |  |  | 0.072 |  |  |  | <0.001 |
| P for interaction |  |  |  |  |  | 0.674 |  |  |  | 0.847 |  |  |  | <0.001 |
| Obesity |  |  |  |  |  |  |  |  |  |  |  |  |  |  |
| BMI<25 | 0 | 1908424 | 7875 | 9683611.14 | 0.81323 | 1 (reference) | 7068 | 9686113.2 | 0.7297 | 1 (reference) | 12473 | 9700349.74 | 1.28583 | 1 (reference) |
|  | 1 | 75200 | 592 | 379760.57 | 1.55888 | 1.15 (1.05,1.25) | 596 | 379822.67 | 1.56915 | 1.26 (1.16,1.38) | 1061 | 381053.14 | 2.78439 | 1.55 (1.45,1.65) |
|  | 2 | 32320 | 296 | 162933.24 | 1.8167 | 1.25 (1.11,1.41) | 270 | 163045.85 | 1.65598 | 1.23 (1.08,1.39) | 489 | 163612.75 | 2.98876 | 1.60 (1.46,1.75) |
|  | 3 | 19060 | 176 | 96159.73 | 1.83029 | 1.18 (1.02,1.38) | 166 | 96183.54 | 1.72587 | 1.20 (1.02,1.40) | 337 | 96527.61 | 3.49123 | 1.82 (1.63,2.03) |
|  | 4 | 11823 | 145 | 59834.52 | 2.42335 | 1.45 (1.22,1.71) | 119 | 59891.06 | 1.98694 | 1.26 (1.05,1.52) | 229 | 60133.39 | 3.8082 | 1.92 (1.68,2.19) |
|  | P for Trend |  |  |  |  | <0.001 |  |  |  | <0.001 |  |  |  | <0.001 |
| BMI ≥25 | 0 | 471626 | 2292 | 2385038.46 | 0.96099 | 1 (reference) | 2101 | 2385739.95 | 0.88065 | 1 (reference) | 2518 | 2389965.69 | 1.05357 | 1 (reference) |
|  | 1 | 135004 | 849 | 680716.34 | 1.24722 | 1.18 (1.09,1.28) | 811 | 680907.2 | 1.19106 | 1.18 (1.08,1.28) | 940 | 682574.74 | 1.37714 | 1.17 (1.08,1.26) |
|  | 2 | 99290 | 650 | 500596.12 | 1.29845 | 1.21 (1.10,1.32) | 680 | 500623.86 | 1.35831 | 1.29 (1.18,1.41) | 741 | 502037.69 | 1.47598 | 1.23 (1.13,1.34) |
|  | 3 | 97058 | 651 | 489267.79 | 1.33056 | 1.21 (1.10,1.33) | 673 | 489202.73 | 1.37571 | 1.26 (1.14,1.38) | 746 | 490659.04 | 1.5204 | 1.24 (1.14,1.36) |
|  | 4 | 153263 | 1103 | 773750.93 | 1.42552 | 1.29 (1.18,1.41) | 1219 | 773548.04 | 1.57586 | 1.37 (1.26,1.50) | 1370 | 776222.5 | 1.76496 | 1.47 (1.35,1.60) |
|  | P for Trend |  |  |  |  | <0.001 |  |  |  | <0.001 |  |  |  | <0.001 |
| P for interaction |  |  |  |  |  | 0.205 |  |  |  | 0.322 |  |  |  | <0.001 |
| Metabolic syndrome |  |  |  |  |  |  |  |  |  |  |  |  |  |  |
| No | 0 | 2074831 | 6434 | 10543435.11 | 0.61024 | 1 (reference) | 6008 | 10545157.23 | 0.56974 | 1 (reference) | 12157 | 10556235.65 | 1.15164 | 1 (reference) |
|  | 1 | 126440 | 574 | 640945.53 | 0.89555 | 1.21 (1.11,1.32) | 574 | 641109.76 | 0.89532 | 1.23 (1.12,1.34) | 1161 | 642104.28 | 1.80812 | 1.54 (1.45,1.64) |
|  | 2 | 66780 | 306 | 338702.73 | 0.90345 | 1.28 (1.14,1.44) | 305 | 338775.21 | 0.9003 | 1.25 (1.11,1.41) | 597 | 339334.96 | 1.75932 | 1.66 (1.53,1.81) |
|  | 3 | 48438 | 208 | 245587.77 | 0.84695 | 1.22 (1.06,1.41) | 206 | 245582.72 | 0.83882 | 1.15 (1.00,1.33) | 438 | 245978.19 | 1.78065 | 1.82 (1.65,2.02) |
|  | 4 | 42943 | 184 | 218289.35 | 0.84292 | 1.34 (1.14,1.56) | 189 | 218331.61 | 0.86566 | 1.23 (1.05,1.43) | 374 | 218646.61 | 1.71052 | 2.19 (1.96,2.44) |
|  | P for Trend |  |  |  |  | <0.001 |  |  |  | <0.001 |  |  |  | <0.001 |
| Yes | 0 | 305219 | 3733 | 1525214.49 | 2.44752 | 1 (reference) | 3161 | 1526695.91 | 2.07048 | 1 (reference) | 2834 | 1534079.79 | 1.84736 | 1 (reference) |
|  | 1 | 83764 | 867 | 419531.38 | 2.06659 | 0.98 (0.91,1.06) | 833 | 419620.11 | 1.98513 | 0.99 (0.91,1.07) | 840 | 421523.6 | 1.99277 | 1.28 (1.18,1.39) |
|  | 2 | 64830 | 640 | 324826.62 | 1.97028 | 1.00 (0.91,1.09) | 645 | 324894.49 | 1.98526 | 1.01 (0.92,1.10) | 633 | 326315.48 | 1.93984 | 1.36 (1.24,1.49) |
|  | 3 | 67680 | 619 | 339839.76 | 1.82145 | 0.98 (0.90,1.09) | 633 | 339803.55 | 1.86284 | 0.98 (0.89,1.07) | 645 | 341208.46 | 1.89034 | 1.46 (1.33,1.60) |
|  | 4 | 122143 | 1064 | 615296.1 | 1.72925 | 1.07 (0.98,1.17) | 1149 | 615107.48 | 1.86797 | 1.04 (0.95,1.13) | 1225 | 617709.27 | 1.98313 | 1.86 (1.70,2.02) |
|  | P for Trend |  |  |  |  | 0.242 |  |  |  | 0.594 |  |  |  | <0.001 |
|  |  |  |  |  |  | <0.001 |  |  |  | <0.001 |  |  |  | <0.001 |
| Abdominal obesity |  |  |  |  |  |  |  |  |  |  |  |  |  |  |
| No | 0 | 2197005 | 8865 | 11152428.66 | 0.79489 | 1 (reference) | 8118 | 11154915.98 | 0.72775 | 1 (reference) | 13540 | 11171362.2 | 1.21203 | 1 (reference) |
|  | 1 | 138793 | 887 | 703293.25 | 1.26121 | 1.15 (1.07,1.24) | 941 | 703274.04 | 1.33803 | 1.25 (1.17,1.34) | 1389 | 705231.86 | 1.96957 | 1.48 (1.40,1.57) |
|  | 2 | 72933 | 500 | 369556.66 | 1.35297 | 1.23 (1.12,1.35) | 505 | 369608.16 | 1.36631 | 1.23 (1.12,1.35) | 713 | 370671.62 | 1.92354 | 1.52 (1.40,1.64) |
|  | 3 | 52034 | 339 | 263839.9 | 1.28487 | 1.16 (1.04,1.30) | 375 | 263742.59 | 1.42184 | 1.24 (1.12,1.38) | 525 | 264567.7 | 1.98437 | 1.66 (1.52,1.82) |
|  | 4 | 42255 | 325 | 214704.82 | 1.51371 | 1.37 (1.22,1.54) | 354 | 214689.24 | 1.64889 | 1.39 (1.24,1.56) | 430 | 215443.84 | 1.99588 | 1.81 (1.64,2.00) |
|  | P for Trend |  |  |  |  | <0.001 |  |  |  | <0.001 |  |  |  | <0.001 |
| Yes | 0 | 183045 | 1302 | 916220.94 | 1.42105 | 1 (reference) | 1051 | 916937.17 | 1.14621 | 1 (reference) | 1451 | 918953.24 | 1.57897 | 1 (reference) |
|  | 1 | 71411 | 554 | 357183.65 | 1.55102 | 1.16 (1.04,1.28) | 466 | 357455.83 | 1.30366 | 1.10 (0.98,1.23) | 612 | 358396.03 | 1.70761 | 1.12 (1.01,1.23) |
|  | 2 | 58677 | 446 | 293972.7 | 1.51715 | 1.17 (1.05,1.31) | 445 | 294061.55 | 1.51329 | 1.27 (1.13,1.43) | 517 | 294978.82 | 1.75267 | 1.18 (1.06,1.31) |
|  | 3 | 64084 | 488 | 321587.63 | 1.51747 | 1.19 (1.07,1.34) | 464 | 321643.68 | 1.44259 | 1.19 (1.06,1.34) | 558 | 322618.95 | 1.72959 | 1.19 (1.07,1.33) |
|  | 4 | 122831 | 923 | 618880.63 | 1.4914 | 1.26 (1.13,1.40) | 984 | 618749.86 | 1.5903 | 1.30 (1.16,1.46) | 1169 | 620912.04 | 1.88271 | 1.43 (1.30,1.58) |
|  | P for Trend |  |  |  |  | <0.001 |  |  |  | <0.001 |  |  |  | <0.001 |
| P for interaction |  |  |  |  |  | 0.103 |  |  |  | 0.022 |  |  |  | <0.001 |
| Diabetes |  |  |  |  |  |  |  |  |  |  |  |  |  |  |
| No | 0 | 2275124 | 8536 | 11546685.69 | 0.73926 | 1 (reference) | 7873 | 11549054.33 | 0.6817 | 1 (reference) | 12705 | 11564901.56 | 1.09858 | 1 (reference) |
|  | 1 | 187791 | 1080 | 949382.9 | 1.13758 | 1.19 (1.11,1.27) | 1088 | 949543 | 1.14581 | 1.22 (1.14,1.30) | 1464 | 951759.51 | 1.5382 | 1.37 (1.29,1.45) |
|  | 2 | 114928 | 671 | 580843.49 | 1.15522 | 1.21 (1.12,1.32) | 720 | 580848.2 | 1.23957 | 1.29 (1.19,1.40) | 887 | 582346.01 | 1.52315 | 1.46 (1.36,1.57) |
|  | 3 | 98940 | 560 | 500141.33 | 1.11968 | 1.18 (1.08,1.29) | 612 | 500015.33 | 1.22396 | 1.24 (1.14,1.36) | 771 | 501321.28 | 1.53794 | 1.59 (1.47,1.72) |
|  | 4 | 134393 | 809 | 680524.62 | 1.18879 | 1.27 (1.16,1.38) | 931 | 680297.44 | 1.36852 | 1.33 (1.22,1.44) | 1019 | 682306.15 | 1.49346 | 1.81 (1.69,1.95) |
|  | P for Trend |  |  |  |  | <0.001 |  |  |  | <0.001 |  |  |  | <0.001 |
| Yes | 0 | 104926 | 1631 | 521963.91 | 3.12474 | 1 (reference) | 1296 | 522798.82 | 2.47897 | 1 (reference) | 2286 | 525413.87 | 4.35086 | 1 (reference) |
|  | 1 | 22413 | 361 | 111094.01 | 3.2495 | 1.11 (0.99,1.25) | 319 | 111186.87 | 2.86904 | 1.15 (1.01,1.31) | 537 | 111868.37 | 4.80028 | 1.42 (1.29,1.57) |
|  | 2 | 16682 | 275 | 82685.86 | 3.32584 | 1.22 (1.06,1.39) | 230 | 82821.5 | 2.77706 | 1.15 (0.99,1.33) | 343 | 83304.44 | 4.11743 | 1.39 (1.24,1.57) |
|  | 3 | 17178 | 267 | 85286.19 | 3.13064 | 1.23 (1.07,1.42) | 227 | 85370.94 | 2.65898 | 1.14 (0.98,1.33) | 312 | 85865.37 | 3.6336 | 1.42 (1.25,1.62) |
|  | 4 | 30693 | 439 | 153060.83 | 2.86814 | 1.35 (1.18,1.55) | 407 | 153141.65 | 2.65767 | 1.25 (1.08,1.45) | 580 | 154049.74 | 3.76502 | 2.09 (1.86,2.34) |
|  | P for Trend |  |  |  |  | <0.001 |  |  |  | 0.003 |  |  |  | <0.001 |
| P for interaction |  |  |  |  |  | 0.030 |  |  |  | <0.001 |  |  |  | 0.002 |

FLI, fatty liver index; HR, hazard ratio; CI, confidence interval; BMI, body mass index

Multivariate model 3 was adjusted for age, sex, smoking, drinking, income, hypertension, dyslipidemia, diabetes, body mass index, and regular physical activity.

**Supplementary Table 2.** Impacts of changes in FLI between the 1^st^ exam and the last exam on outcomes.

|  | 1^ST^ FLI | Last FLI | N | Events | person-years | Incidence Rate per 1000 | Hazard ratio (95% confidence interval) | | | |
| --- | --- | --- | --- | --- | --- | --- | --- | --- | --- | --- |
|  |  |  |  |  |  |  | Univariate model | Multivariate model 1 | Multivariate model 2 | Multivariate model 3 |
| Death | < 60 | < 60 | 2492986 | 16081 | 12662793.64 | 1.26994 | 1(ref.) | 1(ref.) | 1(ref.) | 1(ref.) |
|  |  | ≥60 | 159652 | 1242 | 804199.52 | 1.54439 | 1.22(1.15,1.29) | 1.20(1.13,1.27) | 1.47(1.38,1.56) | 1.46(1.37,1.55) |
|  | ≥60 | <60 | 122380 | 1411 | 621183.56 | 2.27147 | 1.79(1.69,1.89) | 1.22(1.16,1.29) | 1.33(1.26,1.41) | 1.33(1.26,1.41) |
|  |  | ≥60 | 228050 | 2170 | 1154959.56 | 1.87885 | 1.48(1.42,1.55) | 1.30(1.25,1.36) | 1.65(1.56,1.74) | 1.64(1.56,1.73) |
| P for trend |  |  |  |  |  |  | <.0001 | <.0001 | <.0001 | <.0001 |
| MI | < 60 | < 60 | 2492986 | 9928 | 12642796.13 | 0.78527 | 1(ref.) | 1(ref.) | 1(ref.) | 1(ref.) |
|  |  | ≥60 | 159652 | 913 | 802294.75 | 1.13799 | 1.46(1.36,1.56) | 1.43(1.33,1.53) | 1.13(1.05,1.22) | 1.13(1.05,1.21) |
|  | ≥60 | <60 | 122380 | 1055 | 618971.03 | 1.70444 | 2.17(2.04,2.31) | 1.60(1.50,1.71) | 1.27(1.19,1.35) | 1.27(1.19,1.35) |
|  |  | ≥60 | 228050 | 1807 | 1151016.18 | 1.56992 | 2.00(1.90,2.11) | 1.78(1.70,1.88) | 1.24(1.16,1.32) | 1.23(1.16,1.31) |
| P for trend |  |  |  |  |  |  | <.0001 | <.0001 | <.0001 | <.0001 |
| Stroke | < 60 | < 60 | 2492986 | 10935 | 12639426.16 | 0.86515 | 1(ref.) | 1(ref.) | 1(ref.) | 1(ref.) |
|  |  | ≥60 | 159652 | 928 | 802150.29 | 1.15689 | 1.34(1.25,1.43) | 1.42(1.33,1.52) | 1.18(1.10,1.26) | 1.17(1.09,1.26) |
|  | ≥60 | <60 | 122380 | 1048 | 618887.32 | 1.69336 | 1.96(1.84,2.09) | 1.45(1.36,1.54) | 1.18(1.10,1.26) | 1.18(1.10,1.26) |
|  |  | ≥60 | 228050 | 1718 | 1151205.06 | 1.49235 | 1.73(1.64,1.82) | 1.67(1.59,1.76) | 1.23(1.16,1.31) | 1.23(1.15,1.30) |
| P for trend |  |  |  |  |  |  | <.0001 | <.0001 | <.0001 | <.0001 |

FLI, fatty liver index; MI, myocardial infarction

Multivariate model 1 was adjusted for age and sex.

Multivariate model 2 was adjusted for age, sex, smoking, drinking, income, hypertension, dyslipidemia, diabetes and body mass index.

Multivariate model 3 was adjusted for age, sex, smoking, drinking, income, hypertension, dyslipidemia, diabetes, body mass index, and regular physical activity.

**Supplementary Figure Legends**

**Supplementary Figure 1.** Flowchart of the enrollment of the study population


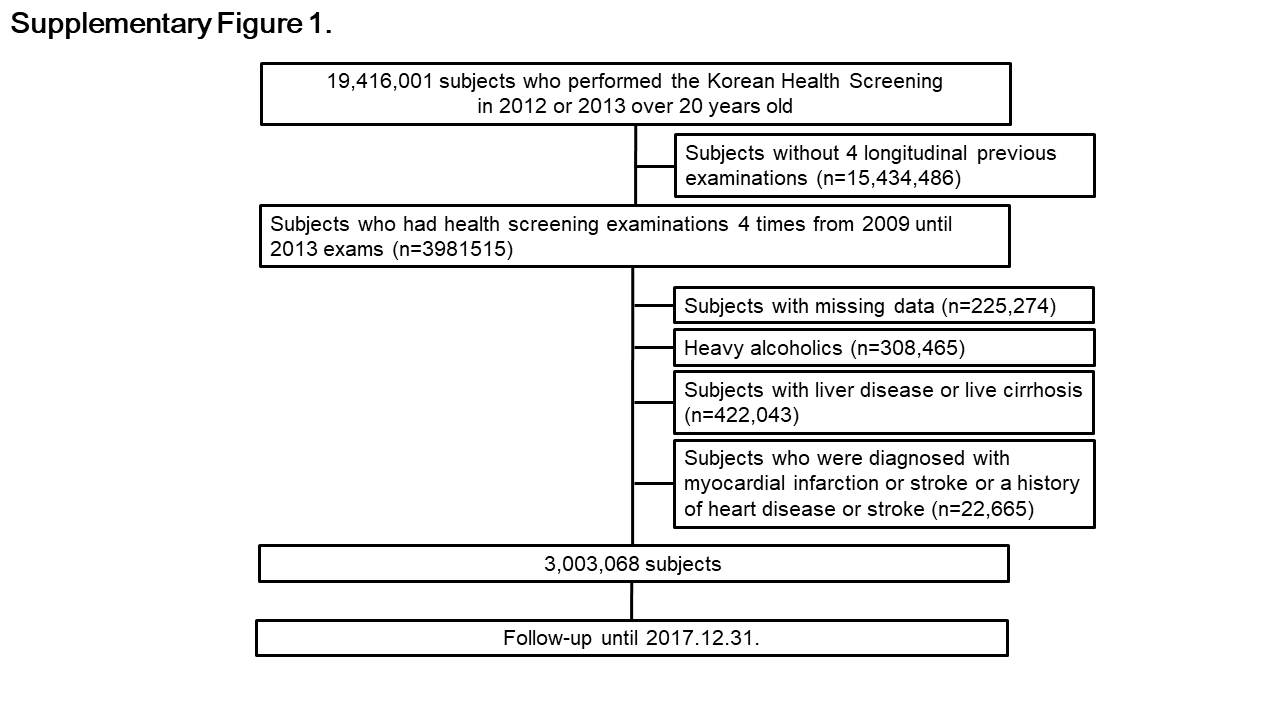

Supplement: Supplementary file 1 [file DataSheet_1.docx]
